# The fecal microbiota of semi-free-ranging wood bison (*Bison bison athabascae*)

**DOI:** 10.1186/1746-6148-10-120

**Published:** 2014-05-28

**Authors:** J Scott Weese, Todd Shury, Murray D Jelinski

**Affiliations:** 1 Department of Pathobiology and Centre for Public Health and Zoonoses, Ontario Veterinary College, University of Guelph, Guelph, ON, Canada; 2 Parks Canada, Saskatoon, SASK, Canada; 3 Department of Veterinary Pathology, Western College of Veterinary Medicine, University of Saskatchewan, Saskatoon, SASK, Canada; 4 Department of Large Animal Clinical Sciences, Western College of Veterinary Medicine, University of Saskatchewan, Saskatoon, SASK, Canada

**Keywords:** Bison, Microbiota, Microbiome, Intestinal

## Abstract

**Background:**

The intestinal tract harbours a complex and diverse microbial population that is important for health, yet has been poorly described in many species. This study explored the fecal microbiota of semi-free-ranging Wood bison (*Bison bison athabascae*).

**Results:**

A total of 2081936 16S rRNA (V4) sequences from 40 bison were evaluated. CatchAll analysis of richness predicted a mean of 10685 species per sample (range 5428–24764, SD 4136). Diversity was high, with an average inverse Simpson’s index of 31.78 (SD 15.3, range 8.55-86.7). Twenty-one different phyla were identified; however, only Firmicutes and Proteobacteria, Actinobacteria accounted for >1% of sequences. Two distinct population clusters (Group A, n = 19 and Group B, n = 21) were evident based on both community membership and population structure. Group A had a significantly lower relative abundance of Actinobacteria (6.4 vs 11.8%, *P* = 0.002), Chloroflexi (0.002 vs 0.013%, *P* = 0.014), Gemmatimonadetes (0.007 vs 0.15%, *P* = 0.038) and Proteobacteria (18.7 vs 42.5%, *P* = <0.0001) and a greater relative abundance of Firmicutes (70.9 vs 39.3%, *P* < 0.0001) than Group B. Within Group B, Alphaproteobacteria was the most common class of Proteobacteria (28% of all sequences), while Caulobacteraceae (18.5%), Pseudomonadaceae (3.5%), Hyphomicrobiaceae (3.5%), Alcaligenaceae (3.1%) and Xanthomonadaceae (2.6%) were the most abundant families.

The twenty (3.1%) most abundant genera accounted for 71% of sequences. No operational taxon units (OTUs) were found in all samples at a relative abundance of 1% or greater. One OTU (*Clostridium* cluster XI) was present at 1% or more in all Group A samples, with two other *Clostridium* cluster XI OTUs in 18/19 (95%) samples. No OTUs were found at that abundance in all Group B sample, but an unclassified Lachnospiraceae was present in 20/21 (95%) and *Clostridium* cluster XI and *Brevundimonas* were found in 19 (90%) samples.

**Conclusions:**

The fecal microbiota of Wood bison is rich and diverse. The presence of two distinct populations not associated with housing, age or gender suggest that enterotypes, distinctly different microbial population compositions that can achieve the same ultimate function, might be present in bison, as has been suggested in humans.

## Background

The American bison (*Bison bison*) is a ruminant herbivore that is found in the wild in regions of western Canada and the United States and also raised in captivity as a food animal. Wood bison (*Bison bison athabascae*) are a geographic variant of Plains Bison (*Bison bison bison*) that are morphologically larger, but not genetically distinct and have historically inhabited areas in northern Canada and Alaska. While they are raised commercially, little is known about the biology and health of bison compared to other domestic food animals. Some information can be inferred from other ruminants, but there are potentially important physiological and microbiological differences [1,2] that indicate the need for specific study.

The gastrointestinal microbial population, the microbiota, is receiving increasing study in many species because of its role in health and various diseases, as well as its complex interaction with the body. Despite the known importance of the intestinal microbiota in many other species, there has been limited study in bison. Culture-based methods have evaluated the fecal and ruminal microbiota of bison [1-4], but it is now clear that such approaches have significant inherent limitations for characterization of the microbiota. Culture-based methods provide a very superficial understanding of the intestinal microbiota because of its complexity, richness (number of different organisms), the fastidious nature of many microorganisms, the potential for profound culture bias (over-representation of bacteria that grow well under typical culture conditions) and because a large percentage of the microbiota is currently unculturable. While not without their own potential biases, culture-independent methods, particularly next generation sequencing, have now become the standard for assessment of complex polymicrobial environments.

Understanding the microbiota of normal animals can be important for assessing the role of the microbiota in disease, as well as the impact of various management practices (e.g. diet change, antimicrobial administration) on the microbiota. Accordingly, the objective of this study was to evaluate the fecal microbiota of members of a semi-free-ranging extensively managed wood bison herd using a culture-independent next generation sequence-based approach.

## Methods

### Study population and sample collection

Fecal samples were collected from 40 semi free-ranging wood Bison, located in a 60 square kilometre fenced area within Elk Island National Park near Edmonton, Alberta, Canada. Bison are fenced behind a 2 metre high fence, but are essentially managed as wildlife within a national park. Animals were a mix of ages and sexes, but were fed the same hay diet for approximately one month prior to sampling. Bison originated from three separate pens in which animals were segregated by age and sex. Pen A housed 7 male calves (approx. 7 months old), pen B housed 20 adult females (>3 years of age) and pen C housed 13 adult males (>3 years of age). None of the animals had any history of antimicrobial exposure within the past two years. Fecal samples were collected per rectum during routine handling in a custom designed facility with alleyways and squeeze chute, which occurs biennially to allow management of surplus animals. Fecal samples were frozen at −20°C prior to analysis. This study was approved by the University of Saskatchewan Research Ethics Office (protocols 20130032 and 20130037).

### DNA extraction and quality control

DNA was extracted from fecal samples using a commercial kit according to the manufacturer’s instructions^a^. DNA quantity and quality were accessed by spectrophotometry^b^.

### 16S rRNA gene amplification and sequencing

The V4 region of the 16S rRNA gene was amplified using the primers forward S-D-Bact-0564-a-S-15 (5′-AYTGGGYDTAAAGNG-3′) and reverse S-D-Bact-0785-b-A-18 (5′-TACNVGGGTATCTAATCC-3′) [5] as has been previously described [6]. PCR products were evaluated by electrophoresis in 2% agarose gel and purified with the Agencourt AMPure XP system.^c^ Sequencing of the library pool was performed at the University of Guelph’s Advanced Analysis Centre using an Illumina MiSeq^d^ and 2×250 chemistry.

### Bioinformatics

The mothur package of algorithms (v1.32.1) was used for analysis [7]. Paired end reads were aligned. Sequences were aligned with the Silva 16S rRNA reference database (http://www.arb-silva.de) [8]. Sequences that were >244 bp or <239 bp in length, contained any ambiguous base calls or long runs (>8 bp) of holopolymers or did not align with the correct region were removed. Chimeras were identified using uchime [9] and eliminated. CatchAll was used to assess species richness [10]. Taxonomy was assigned using the RDP taxonomy database (http://rdp.cme.msu.edu/index.jsp). Sequences were binned into operational taxon units (OTUs) at a 3% dissimilarity level.

Subsampling was performed to normalize sequence numbers for further comparison. This consisted of random selection of a number of sequences from each sample that corresponded to the lowest sequence abundance of all samples. Coverage was assessed using Good’s coverage and visualized using rarefaction curves. Population diversity was described using the inverse Simpson’s index and evenness was assessed using Shannon’s evenness. Community membership was compared using the traditional Jaccard index, while community structure was assessed using the Yue & Clayton measure of dissimilarity. The core microbiota was assessed through identification of OTUs present in all samples at a minimum relative abundance of 1%.

Visual assessment of dendrograms identified two distinct groups, which were studied further. Relative abundances and alpha diversity indices were compared using t-test. The parsimony test was applied to the Jaccard and Yue & Clayton trees. Dissimilarity was also visualized using principal coordinate analysis (PCoA), and the statistical significance of the observed spatial separation was assessed using Analysis of Molecular Variance (AMOVA) and analysis of similarity (ANOSIM). A P value of <0.05 was considered significant for all comparisons.

## Results

A total of 2081936 V4 16S RNA gene sequences from 40 bison passed all quality control filters. The number of sequences per sample ranged from 17667 to 106284 (mean 52049, SD 16896). CatchAll analysis of richness predicted a mean of 10685 species per sample (range 5428–24764, SD 4136). Good’s coverage ranged from 0.943-0.976 (mean 0.962, SD 0.0097). Rarefaction curves based on subsampling of 17667 sequences per sample are displayed in Additional file 1: Figure S1. Diversity was high, with an average inverse Simpson’s index of 31.78 (SD 15.3, range 8.55-86.7). Shannon’s evenness values were an average of 0.60 (SD 0.046, range 0.477-0.683).

**Figure 1:**
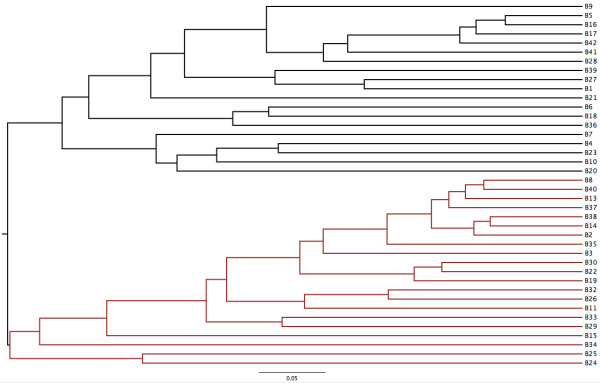
Population structure of the fecal microbiota of forty bison (Black: Group A, Red: Group B).

**Figure 2:**
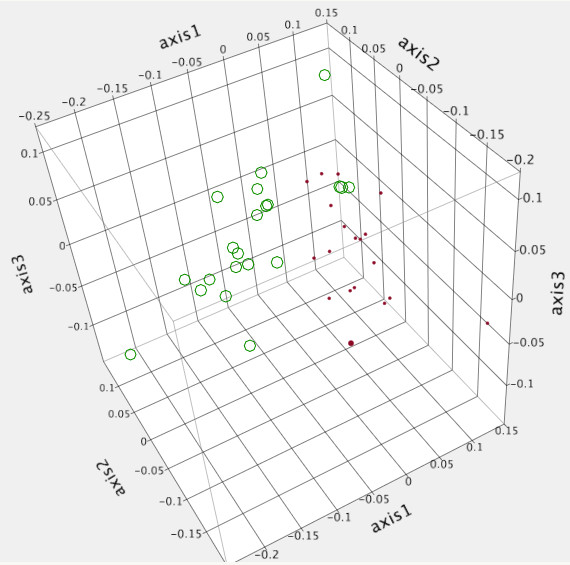
Principle coordinate analysis of the bacterial microbial population of forty bison, separated into two different enterotypes (Group A: red, Group B: green).

Two distinct clusters (Group A, n = 19 and Group B, n = 21) were evident visually with both the Yue & Clayton (Figure 1) and Jaccard (data not shown) dendrograms. This was also evident visually using PCoA (Figure 2). Community membership and structure were significantly different based on parsimony test applied to both the Yue & Clayton (*P* = 0.034) and Jaccard (*P* = <0.001) trees. Significant differences in community structure were also observed with AMOVA (*P* < 0.001) and analysis of similarity (ANOSIM) (*P* < 0.001). There was no difference in evenness between groups (0.644 vs 0.640, *P* = 0.78) and while there was not a significant difference in diversity (inverse Simpson’s, 36.7 vs 27.3) it approached significance (*P* = 0.06). There was no association between pen-of-origin and Group (*P* = 0.53, Table 1).

**Table 1:** Pen classification of two different groups of fecal microbiota in bison

**Pen**	**Description**	**Group A**	**Group B**	**Total**
A	Male calves	2	5	7
B	Adult females	10	10	20
C	Adult males	7	6	13
Total		19	21	40

**Figure 3:**
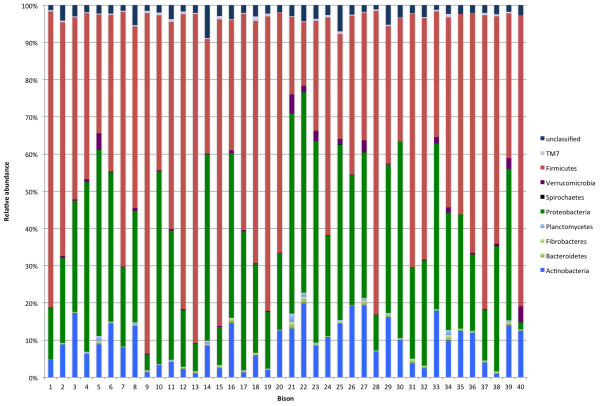
Relative abundance of the predominant bacterial phyla in the feces of 40 bison.

Twenty-one different phyla were identified (Table 1, Figure 3); however, only three (Firmicutes, Proteobacteria, Actinobacteria) accounted for >1% of sequences overall. There were numerous significant differences in relative abundances of different phyla between groups (Table 2), with the most pronounced differences being with Firmicutes and Proteobacteria. Group A had a significantly lower relative abundance of Actinobacteria (6.4 vs 11.8%, *P* = 0.002), Chloroflexi (0.002 vs 0.013%, *P* = 0.014), Gemmatimonadetes (0.007 vs 0.15%, *P* = 0.038) and Proteobacteria (18.7 vs 42.5%, *P* = <0.0001) and a greater relative abundance of Firmicutes (70.9 vs 39.3%, *P* = <0.0001) than Group B. Six hundred fifty two genera were identified, with *Clostridium* cluster XI the most abundant overall (13%), followed by *Brevundimonas* (9.4%). The predominant genera in each group are presented in Table 3.

**Table 2:** Relative abundance of bacterial phyla found in the fecal microbiota of forty bison, divided into two different groups

**Phylum**	**Group A (n = 19)**	**Group B (n = 21)**	**P value**
Acidobacteria	0.00008	0.00004	0.43
**Actinobacteria**	**6.4**	**1.2**	**0.002**
Bacteroidetes	0.49	0.65	0.26
Chlamydiae	0.018	0.036	0.24
**Chloroflexi**	**0.0029**	**0.013**	**0.014**
**Deferibacteres**	**0**	**0.00005**	**0.033**
Deinococcus-Thermus	0.00004	0.0023	0.40
Elusimicrobia	0.000013	0.000008	0.72
Fibrobacteres	0.016	0.018	0.80
Fusobacteria	0.0029	0.0022	0.67
Gemmatimonadetes	0.007	0.15	0.038
Lentispaerae	0.0028	0.00005	0.24
Plantomycetes	0.15	0.42	0.065
**Proteobacteria**	**18.7**	**42.5**	**<0.0001**
Spirochaetes	0.035	0.022	0.35
Synergistetes	0.0001	0.0018	0.24
Tenericutes	0.0015	0.0030	0.15
Verrucomicrobia	0.54	1.3	0.056
**Firmicutes**	**70.9**	**39.3**	**<0.001**
SR1	0	0.000055	0.11
TM7	0.42	0.42	0.97
Unclassified	2.3	3.3	0.06

Phyla that were significantly different area indicated in bold.

**Table 3:** Relative abundance of predominant genera in the fecal microbiota of 40 bison

**Genus**	**Total**	**Group A**	**Group B**	***P *** **value**
*Clostridium* cluster XI	13.1%	17.5%	8.3%	0.004
*Brevundimonas*	9.5%	4.3%	15.3%	<0.001
Unclassified Clostridiaceae	8.9%	10.1%	7.4%	0.013
Unclassified Lachnospiraceae	6.6%	7.8%	5.1%	0.023
*Sedimentibacter*	4.2%	5.9%	1.9%	0.001
*Pseudomonas*	3.4%	3.4%	2.5%	0.56
Unclassified Peptostreptococcaceae	2.9%	3.9%	1.7%	0.014
Unclassified bacterium	2.8%	2.4%	3.3%	0.055
*Tissierella*	2.4%	3.0%	1.9%	0.16
*Devosia*	2.2%	1.1%	3.3%	0.0007
Unclassified Clostridia	1.7%	2.0%	1.5%	0.065
Unclassified Comamonadaceae	1.6%	1.3%	1.7%	0.55
*Nocardioides*	1.5%	1.1%	2.3%	0.028
*Phenylobacterium*	1.5%	0.5%	2.9%	0.013
*Pusillimonas*	1.5%	1.0%	1.9%	0.006
Unclassified Ruminococcaceae	1.4%	1.9%	0.8%	0.002
*Sporomusa*	1.4%	2.5%	0.7%	0.008
*Aeromicrobium*	1.3%	0.9%	1.8%	0.18
*Carnobacterium*	1.3%	1.5%	0.9%	0.076
Unclassified Bacillales	1.2%	1.5%	1.0%	0.020

Within Group B, Alphaproteobacteria was the most common class of Proteobacteria (28% of all sequences) followed by Gammaproteobacteria (8.0%) and Betaproteobacteria (6.0%). Caulobacterales (18.5%), Rhizobales (7.4%), Burkholderiales (5.9%), Pseudomonadales (3.9%) and Xanthomonadales (1.6%) were the main orders, while Caulobacteraceae (18.5%), Pseudomonadaceae (3.5%), Hyphomicrobiaceae (3.5%), Alcaligenaceae (3.1%) and Xanthomonadaceae (2.6%) were the most abundant families.

A total of 652 different genera were identified. Most were rare, with the twenty (3.1%) most abundant genera accounting for 71% of total sequences. No OTUs were found in all samples at a relative abundance of 1% or greater. One (*Clostridium* cluster XI) was found at a relative abundance of at least 1% in 38/40 (95%) samples, two (*Clostridium* cluster XI and an unclassified Lachnospiraceae) in 37/40 (93%) samples and 4 (two *Clostridium* cluster XI, unclassified Lachnospiraceae, *Brevundimonas*) in 33/40 (83%) samples.

When Group A was analysed separately, one OTU (*Clostridium* cluster XI) was present at 1% or more in all samples, with two other *Clostridium* cluster XI OTUs were present in 18/19 (95%) samples. No OTUs were found at that abundance in all Group B sample, but an unclassified Lachnospiraceae was present in 20/21 (95%), while a *Clostridium* cluster XI and *Brevundimonas* were found in 19 (90%) samples.

## Discussion

The fecal microbiota of bison contains a rich and diverse microbial population. It was rather surprising to find two distinct subpopulations since animals harbouring these subpopulations had been co-housed and fed the same hay-based diet for approximately one month. Typical factors associated with gut microbiota alteration such as differences in diet, management, diet change, age, antimicrobial administration and gastrointestinal disease were not present, there was no association with age or gender, and no other differences between these groups were evident. Reasons for this are unclear but one potential relates to the concept of ‘enterotypes’. It has been proposed that the intestinal microbiota of most humans can be categorized into three enterotypes based on predominance of different bacterial groups, *Bacteroides, Prevotella* or *Ruminococcus*[11]. The basis behind this concept is that different population structures may be able to achieve similar function in the intestinal tract; however, it is unclear whether truly distinct enterotypes exist [12] and, if so, the relevance. Enterotypes have not been reported in other species, yet the presence of markedly different fecal microbiotas amongst similar animals in this study suggests this phenomenon might occur in bison. This could be further explored by study of the functional genetic composition of the microbiota rather than relying solely on phylogeny.

The clinical relevance of the group differences noted here is unclear. Consistent with the concept of enterotypes, it is possible that there is adequate functional redundancy so that even profound differences in microbial populations can achieve the same functional results in the intestinal tract. However, study of the potential health impacts of these different microbial populations is warranted. This is perhaps most relevant in Group B, considering the high prevalence of Proteobacteria and relatively low abundance of Firmicutes. Proteobacteria tend to be of much lower abundance in the feces of healthy ruminants and other species compared to the relative abundance noted here [13-16], and increases in Proteobacteria are often associated with ‘dysbiosis’ and intestinal inflammation [17-19]. Increases in the relative abundance of Proteobacteria have been reported with various types of intestinal tract disease in diverse species such as humans, horses and dogs [13,14,18,20]. This study cannot evaluate the health impacts of Proteobacteria or other groups in bison, but the striking differences and concern about this phylum in other species raise some questions that should be addressed further. In contrast, the high abundance of Firmicutes in Group A is consistent with previous reports in ruminants [15,16,21] and hindgut fermenters [14,22]. This phylum consists of a broad range of Gram positive bacteria with diverse functions and clinical relevance. It includes a large number of species with important roles in digestion and energy conversion and is expected to be present in large numbers.

The dominance of a small number of phyla is consistent with other fecal microbiota studies, where a large number of phyla may be present with only a few accounting for more than 1% of sequences. Typically, Firmicutes, Bacteroidetes, Proteobacteria, Actinobacteria and Verrucomicrobia are most common [14,23-26], as was the case here. The relative abundance of Bacteroidetes was low compared to studies of cattle [15,16,21]; however, care must be taken when making such comparisons and comparison of bison and cattle samples using the same laboratory and analytical methods is required to evaluate potential differences between those species.

Three percent of genera accounted for 71% of sequences, yet no OTUs were found in all samples at a minimum relative abundance of 1%. Significant differences between the two Groups for most of the main genera may be one explanation of the lack of an identified core microbiota between all bison. One *Clostridium* cluster XI OUT was the closest to a true core component, being found in all Group A samples and 90% of Group B samples as 1% of more of the sequences. *Clostridium* cluster XI has been reported to be a common constituent of the fecal microbiota of a diverse range of species, including dogs, cats, pigs, grizzly bears and humans [27-30], although it has received limited investigation. It has been suggested that *Clostridium* cluster XI levels may correlate with dietary protein in carnivores; [29], however, this group also includes many saccharolytic organisms [31], something that might account for the relatively high abundance in hay-fed bison. It is likely that this is a very broad group from a functional standpoint, with individual members that can play different roles in diverse gastrointestinal environments.

While this study can define microbial evenness and diversity, ideal values are not known so interpretation of these results in a clinical context is difficult. Greater diversity likely provides an added degree of functional redundancy, whereby there is a functional ‘reserve’ capacity in the microbiota to adapt to external influences. Yet, optimal diversity is unknown and some degree of unevenness (greater representation of some members of the microbiota) is expected, since some bacteria play more important roles than others (e.g. cellulolytic organisms should probably be present at greater abundances than many other species in a herbivore).

Comparison with previous culture-dependent study highlights the different results that can be obtained. One study of 96 bison samples involved a comprehensive culture-based approach yet only identified 19 ‘major’ genera and a small but unclear number of lesser genera [4]. In contrast, 228 genera were identified in the current study, with species estimates exceeding 10000 per sample. Relative abundance was not evaluated in that study, but none of the ten most prevalence genera in the culture-dependent study were amongst the most abundant genera identified here (Table 3). Rather than true differences in populations, these contrasts likely reflect the difficulty growing many of the common genera identified here by sequence-based methods and overgrowth-bias of potentially uncommon genera that are adept at growing under conventional culture conditions.

Fecal analyses must always be evaluated with an understanding that there are differences in the microbiota of different parts of the intestinal tract. This may be particularly important in bison, as the proximal location and markedly different environment of the rumen mean that feces do not closely reflect the state of the ruminal microbiota [16]. Yet, while these differences may limit the sensitivity of the use of feces for detection of alterations in proximal compartments, it is reasonable to assume that differences in the fecal microbiota indicate differences in at least some proximal locations, and that the intestinal (post-ruminal) microbiota is also important. Comparative study of the entire gastrointestinal tract can be informative [16] but requires either surgical collection of samples or collection immediately after euthanasia, both of which carry inherent logistical, cost and ethical issues. Concurrent study of the rumen and feces is more practical because rumen fluid collection is more feasible (although certainly not without challenges in bison). Future study comparing the rumen microbiota between different bison, and comparing the rumen and fecal microbiota would be useful. Further, this study also only involved one population of bison, so extrapolation to other populations of wild or captive bison, particularly those under different management and from different regions, must be done with care. Study of wild and captive bison populations from different regions would be useful to compare with the results obtained here and to help determine the ‘normal’ microbiota and its variations.

## Conclusion

Two distinct microbial populations were identified in a group of semi-free-ranging bison, with no apparent explanation for the differences. Numerous bacterial phyla were identified, yet a small number accounted for the vast majority of the microbiota. Further study of the potential that distinct enterotypes may exist in the bison fecal microbiota, and if so, the clinical implications of such as phenomenon, is indicated to further explore the potential impact of this on bison health.

## End notes

^a^E.Z.N.A. Stool DNA Kit, Omega Bio-Tek Inc., Doraville, GA, USA.

^b^NanoDrop, Roche, Mississauga, Canada. 

^c^Beckman Coulter Inc, Mississauga, Ontario, Canada.

^d^Illumina, San Diego, CA, USA.

### Availability of supporting data

The dataset supporting the results of this article is available at the MG-RAST metagenomics analysis server (project TBA, http://metagenomics.anl.gov).

## Competing interest

The authors declare that they have no competing interests.

## Authors’ contribution

JSW, TS and MJ designed the study. TS coordinated fecal collection. JSW directed laboratory testing, performed data analysis and prepared the manuscript. All authors read and approved the final manuscript.

## Supplementary Material

Additional file 1: Figure S1 Rarefaction curves from assessment of the fecal microbiota of forty bison. Click here for file
