# Supplementary figures and images for: The fecal microbiota of semi-free-ranging wood bison (Bison bison athabascae)
Source: BMC Vet Res. 2014 May 28;10:120. doi: 10.1186/1746-6148-10-120 (PMC4048625; doi:10.1186/1746-6148-10-120)

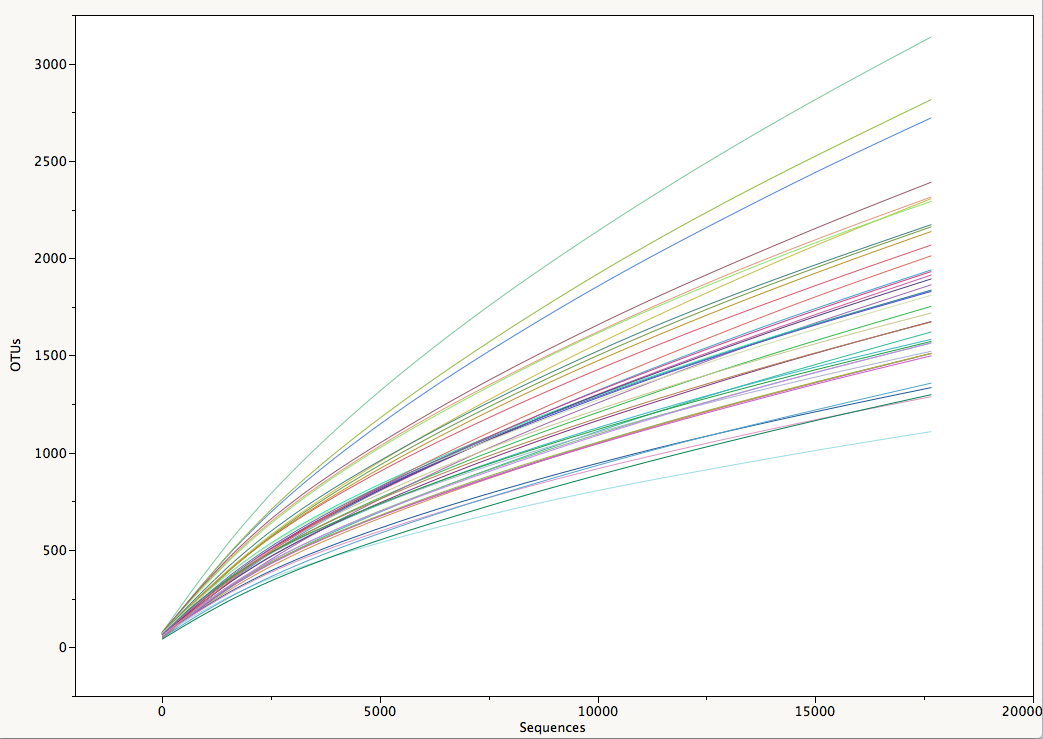

Supplement: Additional file 1: Figure S1 — Rarefaction curves from assessment of the fecal microbiota of forty bison. [file 1746-6148-10-120-S1.tiff]
